# Remote Sensing of Agro-droughts in Guangdong Province of China Using MODIS Satellite Data

**DOI:** 10.3390/s8084687

**Published:** 2008-08-08

**Authors:** Maofang Gao, Zhihao Qin, Hong'ou Zhang, Liping Lu, Xia Zhou, Xiuchun Yang

**Affiliations:** 1 Institute of Agricultural Resources and Regional Planning, Chinese Academy of Agricultural Sciences, Beijing 100081, China; 2 Guangdong Public Laboratory of Environmental Science and Technology, Guangzhou Institute of Geography, Guangzhou 510650, China; 3 International Institute for Earth System Science, Nanjing University, Nanjing 210093, China

**Keywords:** Guangdong province, agricultural drought, drought monitoring, remote sensing, MODIS data

## Abstract

A practical approach was developed in the study for drought monitoring in Guangdong province of China on the basis of vegetation supply water index (VSWI) and precipitation distance index (PDI). A comprehensive index for assessment of agro-drought severity (SADI) was then established from the normalized VSWI and PDI. Using MODIS satellite images and precipitation data from ground-observed meteorological stations, we applied the approach to Guangdong for drought monitoring in 2006. The monitoring results showed that the drought severity on average was very low in the province during the main growing season from May to September in 2006. However, seasonal variation of the severity was also obvious in difference counties of the province. Higher severity of drought could be seen in the periods of late-June (In China each month is traditionally divided into 3 periods. Each is with 10 days and has different names. This division system is mainly with consideration of farming seasons hence has been widely used as the basis of drought monitoring periods in China. In order to keep this tradition, we define, for example, for June, the early-June as the period from 1^st^ to 10^th^ of June, the mid-June as the period from 11^th^ to 20^th^, and the late-June as the period from 21^st^ to 30^th^. So mid-August denotes the period from 11^th^ to 20^th^ of August, and early-July the period from 1^st^ to 10^th^ of July, and so on.), early-July, mid-August and late-September. Regionally, Leizhou Peninsula in the west had the most serious drought before mid-May. Validation indicated that our monitoring results were generally consistent with the drought statistics data and the results from Chinese National Satellite Meteorological Center (CNSMC), which used only remote sensing data. This consistence confirmed the applicability of our approach for drought monitoring. Our better identification of drought severity in Leizhou Peninsula of western Guangdong than that of CNSMC might suggest that the approach developed in the study was able to provide a better alternative to increase the accuracy of drought monitoring for agricultural administration and farming.

## 1. Introduction

Agriculture is an important economic sector in Guangdong province of China, where precipitation distributes unevenly in both spatial and temporal dimensions. More than 60% of annual rainfall drops in the short period from June to August, leading to frequent occurrence of agro-drought in other months of the year. Seasonal droughts usually occur in winter and spring to shape a significant impact on agricultural harvest. Drought monitoring has thus been a necessary effort to alleviate the impact. Generally drought can be monitored through either ground observation or remote sensing. Ground observation is a direct and accurate way for drought monitoring but relatively slow to get enough information for the monitoring in the whole region. It also needs a lot of labor to obtain the necessary information, hence is also expensive. As a contrast, remote sensing from space represents a fast and economic way for the monitoring, but needs to elaborately develop an applicable approach for the region under study.

There were two main approaches to monitor agro-drought with remote sensing data. The first one was based on the assumption that changes of soil moisture under drought would lead to significant changes of soil spectral reflectance observed on remote sensing images. Bowers and Hanks (1965) found that the increase of soil humidity in bare surface would lead to the decrease of soil reflectivity [1]. Thus they suggested a linear correlation between soil moisture and the moisture's absorption bands for remote sensing of soil moisture intensity [2]. The famous thermal inertia model proposed by Watson et al. (1971) had been successfully applied to discriminate geological units according to soil properties (Watson et al. 1974) [3-4]. Pratt and Ellyett (1979) [5] altered the thermal inertia model for soil moisture mapping and Price (1985) [6] improved the model using apparent thermal inertia concept on the basis of surface energetic balance theory. This approach was usually suitable for regions with bare land and sparse vegetation.

The second approach was based on the assumption that drought would lead to the change of vegetation in its physiological process, which consequently affected the spectral attribute of vegetation leaves under observation of remote sensing. Idso et al. (1975, 1981) and Jackson et al. (1981) applied the approach for drought monitoring through the concept of crop water stress index [7, 8, 9]. In recent decades several indices, such as vegetation condition index (Kogan 1990) [10]; temperature-vegetation drought index (Sandholt 2002) [11], and vegetation supply water index (Carlson et al. 1990, 1994) [12,13] were proposed under this approach for drought monitoring on the basis of vegetation index and land surface temperature. Though these indices were generally suitable for the regions with dense crop canopy cover, problems still remained. The value of these indices changed not only in different places but also in different growing seasons for a specific cropping region. Moreover, the relationship between the value and the drought severity is not direct but conditional. The same value of the indices might not mean the same drought severity, while different values might have the same drought severity. This made the determination of drought severity grades according to the value of the indices different with locality. Thus it is very necessary to develop a suitable approach for each region under monitoring.

It was with this consideration that the objective of present study was to develop an applicable approach for agro-drought monitoring in Guangdong. A case study of drought monitoring in 2006 was also selected for validation of the approach and examination of drought sequence in the province. Though several index methods were available for drought monitoring, they were not oriented to Guangdong where paddy rice and subtropical orchards were the main cropping systems and seasonal variation of precipitation is very high. Combination of these two characteristics led to the serious impacts of seasonal droughts on agriculture in the province. Development of an applicable approach for agro-drought monitoring was urgently required for better administration of agricultural farming in the province. The approach might also be applicable to other provinces with similar cropping systems and climate features in south China.

## 2. Methodology

### 2.1. The study region

**Figure 1.**
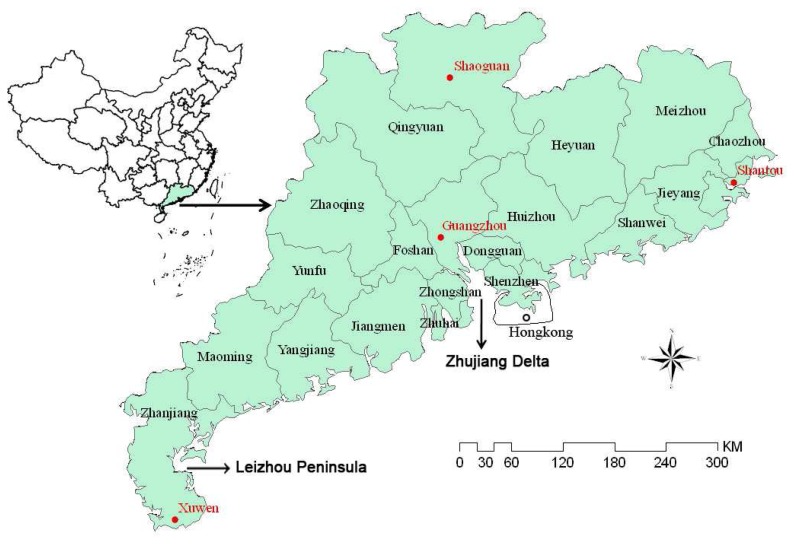
Geographical location of Guangdong province in China, with four meteorological stations mentioned in section 3.

Guangdong province locates in the south of China with complex geomorphology (Figure 1). Nanling Mountain situates in the north with the highest peak of 1903m. The elevation is lower in the south part with inter-valley plains and mounts. Climatologically the province crosses the tropical and subtropical zones from south to north, and has plenty of light, heat and rainfalls. However, influenced by monsoon climate, seasonal distribution of precipitation was uneven in the province. Precipitation in the wet season ranging from April to September accounted for over 80% of the annual total. High variation among years was another distinct feature of rainfall in the province. Widespread of Karst landscape made storage of water from leaking uneasy for compensation of uneven rainfall. The popular red soil in the province also has low capacity of water storage.

Drought disaster often happened in Guangdong as a result of both inhomogeneous precipitation distribution and special landscape structure. Mountains in the north blocked perceptible clouds transporting from north China to form precipitation in spring season, making spring drought aggravating from north to south. Leizhou Peninsula had more drought events because of low precipitation, probably attributed to its low altitude unfavorable to terrain rainfall. Drought in 2004 was very severe in the province to affect 728 thousand hectares of croplands with direct economic loss of RMB 35 billion yuan (USD 1$=RMB 7 yuan). Dynamic monitoring of drought had been recognized as an important measure to ensure the stable agricultural development in the province. The monitoring could provide local administrations usual information of drought events in both spatial distribution and temporal process so that decision making of anti-drought campaigns could be formulated to alleviate the impacts of drought on agricultural development.

### 2.2 Image data for the study

Due to free accessibility and daily availability, MODIS satellite data was used in the study for agro-drought monitoring. With two platforms (Terra and Aqua, respectively), MODIS had 2-4 daytime images per day for the same region, which was very suitable for dynamic monitoring of agro-drought events. Over 1,500 MODIS images of the year 2006 obtained from both Beijing and Guangzhou MODIS ground receiving stations were used to examine the sequence of agro-drought in Guangdong. The data were used to calculate vegetation supply water index (VSWI) as drought indicator. Ten-day composite was made on the index for analysis of drought sequence. Composite was then combined with ground-based meteorological observation data and basic GIS data to obtain drought consequential mapping during the main cropping seasons from May to September in 2006.

### 2.3 Vegetation index for the agro-drought monitoring

Vegetation index was an important parameter in calculating VSWI for drought monitoring. Since green vegetation had strong absorption of spectrum in red region and high reflectance in infrared region, vegetation index was thus generally formulated as various combinations of red and infrared bands. Over 20 indices developed as vegetation index and the most famous was the normalized difference vegetation index (NDVI), hence would also be used in this study for computation of VSWI. For MODIS data, NDVI can be calculated as:

(1) $$\text{NDVI}=\frac{\rho_{2}-\rho_{1}}{\rho_{2}+\rho_{1}}$$

where ρ_1_ and ρ_2_ were reflectance in MODIS bands 1 and 2 respectively. Since MODIS used on-orbit calibration method for its data, implying that the calibration parameters would vary with times and channels, it was necessary to acquire the essential calibration parameters, scales and offsets, from the header file of each image. Thus reflectance in Eq.(1) could be calculated as follows (NASA 2003) [14]:

(2) $$\rho_{ij}=(DN_{ij}-\text{offset}t_{i})\cdot {\text{scales}}_{i}$$

where ρ_ij_ was the reflectance of MODIS band *i* (*i*=1 or 2) for pixel *j*, *DN_ij_* was the digital number of band *i* for pixel *j*, *offset_i_* and *scale_i_* were the offset and gain of band *i*, respectively, obtained from the header file of MODIS image data.

As well known, cloud was usually a big obstacle in application of remote sensing data for drought monitoring because the ground information of cloud pixels was difficult to reveal. It was necessary to detect and remove the influence of cloud in remote sensing of agro-drought. Cloud detection had been done in the study using the standard algorithm of NASA (2003) [14], which classified image pixels into four types according to cloud states: confident cloudy, probably cloudy, probably clear and confident clear. Only pixels with the last two types were used to calculate VSWI for drought monitoring.

### 2.4 Land surface temperature retrieval for drought monitoring

Land surface temperature (LST) was also an essential parameter to calculate VSWI for agro-drought monitoring. In spite of initially designed for NOAA/AVHRR data, the two-factor model of split window algorithm proposed by Qin et al. (2002) [15] was a good alternative for LST retrieval from thermal remote sensing data with two adjacent bands because the model had a comparatively high accuracy and only requires two essential parameters in its computation. Recently, Qin et al. (2005) [16] alternated the algorithm to adapt the thermal bands of MODIS data for LST retrieval. Spectral range of MODIS bands 31 and 32 was closed to that of AVHRR channels 4 and 5. Thus, LST retrieval from MODIS data was done through the use of the two thermal bands. Ground emissivity and atmospheric transmittance were the two essential parameters for LST retrieval from MODIS data and methods had also been developed in Qin et al. (2005) [16] for the estimate of the two parameters from the same scene of MODIS image. Therefore the algorithm of Qin et al. (2005) [16] was very suitable for LST retrieval from MODIS data for drought monitoring and was used in the study for analysis of drought sequence in Guangdong. Formula of the algorithm was not given here for limitation of volume and details could be found in Qin et al. (2002, 2004) [15, 16].

### 2.5 Vegetation supply water index (VSWI) as indicator of agro-drought severity

Growing crops need continuous supply of soil water to ensure harvest. Rainfall and irrigation were main sources of soil water in agricultural fields. When soil water supply was sufficient for crop growing, evapotranspiration from agricultural fields would be high, leading to low surface temperature observed in satellite remote sensing images. During the drought period, soil water supply was in shortage to meet the normal demand of crop growing. Consequently stoma on crop leaves tends to close in order to decrease water lost from canopy, leading to apparent increase of temperature in the fields. Therefore, using the relationship between canopy temperature change and soil water supply in the fields, we were able to develop an approach for drought monitoring. VSWI developed in Carlson et al. (1990, 1994) [12, 13] had been widely applied as drought indicator under the philosophy of this approach. The VSWI related variation of canopy temperature to vegetation index of crops in the following formula for drought monitoring:

(3) $$\text{VSWI}=\frac{\text{NDVI}}{Ts}$$

where *T_s_* was crop canopy temperature in the fields, which could be perceived as LST retrieved from remote sensing images.

Since the VSWI was simple and easy to compute, it had been widely applied to studies of soil moisture and drought monitoring. However, the relationship between VSWI and drought severity was complex or not direct. The increase of VSWI might not surely imply an increase of drought severity in the region under study. This was especially true in the case where crop was in its early growing when canopy did not fully cover the ground. Therefore, the approach of directly applying VSWI to drought monitoring still remained problems and establishment of relationship between VSWI and drought severity needed to be improved in order to make it suitable for purpose of our drought monitoring in Guangdong province.

### 2.6 Improvement of VSWI for agro-drought identification

As well known, crop NDVI was relatively steady within a short time such as one day, while crop canopy temperature may changed remarkably in a day, hence with a feature of diurnal cycling as a result of solar radiation. This implied that *T_s_* in Eq. (3) changed remarkably between day and night. Consequently the value of VSWI changed in accordance with *T_s_*, providing higher possibility of *T_s_* in determination of drought severity when VSWI was used to indicate the severity. On the other hand, *T_s_* had a complicated relation with terrain properties of ground surface. For example, northern Guangdong was dominated with mountainous landscape while the southern part was with plains and low hills. Different land surfaces might lead to high differences of crop canopy temperature *T_s_* in computing VSWI, which may consequently caused bias in indicating the actual severity of agro-drought. In order to solve the problem of VSWI in directly indicating drought severity, extensive discusses had been carried out in this study to analyze the effects of changes in *T_s_* and vegetation indices on VSWI and its relation with drought severity for possibility of improvement.

After several efforts of experiments, we discovered that the improvement with consideration of vegetation fraction in relating VSWI to drought severity could produce the best capability of indicating the severity with VSWI. To perfect the approach, we proposed the following formula to normalize VSWI for scaling drought severity (Qin et al. 2005) [17]:

(4) $$\text{SDI}=(\text{VSWI}‐{\text{VSWI}}_{d})/({\text{VSWI}}_{w}‐{\text{VSWI}}_{d})*100\%$$

where SDI was the normalized VSWI with values ranging from 0 to100 (SDI=0 as the severest drought and *SDI*=100 as the wettest condition), *VSWI_d_* and *VSWI_W_* denoted the values of *VSWI* in the two extremities respectively: the severest drought and the wettest condition. Since vegetation fraction or density could be generally indicated by NDVI, we used the NDVI as a measure to scale the grade of drought severity in the study. Therefore, determination of the values of VSWI for *VSWI_d_* and *VSWI_W_* could be done as followings. Supposed that the step of NDVI was *d* when the distribution of NDVI was from *n* to (*n*+*d*) and the temperature favorable for crop growing was from *T*_1_∼*T*_2_, we could get *VSWI_d_*=(*n*+*d*)/*T*_2_ and *VSWI_W_*=(*n*+*d*)/*T*_1_. For example, the step of NDVI was usually 0.05. In Guangdong where subtropical climate was dominated, the favorable temperature for crop growing was usually recognized as ranging from 20°C to 45°C. Thus, the values of *VSWI_d_* and *VSWI_W_* could be determined as those shown in Table 1.

### 2.7 Precipitation distance index for drought monitoring

Drought in a region was generally a complicated process with multiple drivers. In addition to NDVI and LST extractable from remote sensing images to indicate drought severity, precipitation was another key factor that drove the process of drought in agricultural region. With this consideration, we proposed an approach to combine remote sensing analysis with precipitation indication for drought monitoring in the study (Qin et al. 2005) [17]. In order to perform this approach, rainfall data in 2006 were collected for 17 meteorological observation stations in Guangdong and its adjacent provinces such as Fujian, Jiangxi, Hunan, Guangxi, and Hainan. At the same time, due to availability, historical data of rainfall for the period 1971-2000 were also obtained for the stations. With these historical data, we calculated the average rainfall for each 10-days period within a year to observe the general variation of soil water supply for cropping ecosystem in Guangdong, which was required to compare with rainfall in the period under study to indicate the drought severity. Meteorologically the concept of precipitation distance index (PDI) had been extensively used to indicate aridity for a period. The index PDI was traditionally computed as the ratio of rainfall in a period to the average rainfall in historical records. In the study we altered this concept for drought monitoring by assuming that an agricultural ecosystem will have enough soil water supplies when the rainfall was double over the historical average. This was true in Guangdong where a monsoon climate brings lots of rainfall during the growing season. However, in arid regions or in dry seasons, the historical average of rainfall might be too small to meet the minimal requirement of crops' normal growing. In these cases, an arbitrary determination of a minimal average rainfall (such as 20mm) was set for convenience of computation. With these considerations, we computed the PDI as follows to determine the dryness in the period under study for drought monitoring (Qin et al. 2005) [17]:

(5) $$\text{PDI}=\frac{R}{2R_{w}}\times 100\%$$

where *R* was rainfall in the period under study (the 10-days period in 2006 in our study), *R_W_* was historical average rainfalls for the same period. Standardization of PDI was necessary to integrate with remote sensing indicator SDI for drought identification. Thus we had *PDI*=0 when *R*=0 and *PDI*=100 when *R*>2*R_W_*.

**Figure 2.**
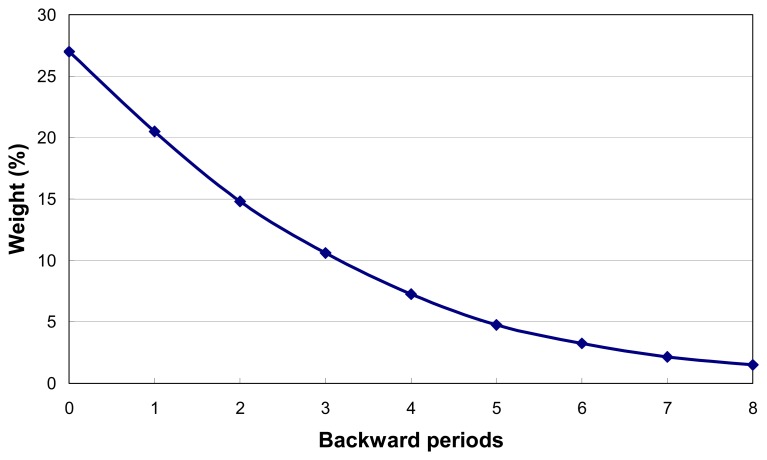
Determination of weights for each backward period in Eq. (6).

On the other hand, relation of precipitation with drought was not direct. A region might not be under drought attack even there was no drop of rainfall in the current period but if it had enough rainfalls in previous periods. This was because drought was a phenomenon resulted from shortage of soil water supply for a long time or several 10-days periods. Actually drought developed as a gradual process of ground drying, which implied that the influence of precipitation on agro-drought was accumulative. Water deficiency within present 10-days period did not ensure the occurrence of drought event in the region. Therefore, we had to consider the rainfalls in the past periods when applying the concept of PDI in identification of drought severity. Our investigation revealed that drought events usually occurred when there was no rain within a period of two months. In this case, we proposed the weighting approach as follows to involve the concept of PDI into our assessment of drought severity (Qin et al. 2005) [17]:

(6) $$\text{MPDI}=A_{0}\times {\text{PDI}}_{0}+A_{1}\times {\text{PDI}}_{1}+A_{2}\times {\text{PDI}}_{2}+A_{3}\times {\text{PDI}}_{3}+\ldots .+A_{8}\times {\text{PDI}}_{8}$$

where MPDI was the improved precipitation distance index for drought assessment, with values ranging from 0 to 100; *PDI_i_* and *A_i_* are PDI and its weight for the period *i* (*i*=0,1,2,…,8), with *i*=0 as present period and *i*=8 as the 8^th^ backward period. The weight of each backward period could be determined according to their importance to formulation of drought. And in the study we determined the values of *A*_1_ to *A*_8_ as shown in Figure 2. Spatial interpolation was required to integrate the MPDI with SDI for agro-drought monitoring and this was done under GIS tool for Guangdong using the MPDI values from the available 17 stations.

### 2.8 Development of approach for agro-drought monitoring in Guangdong

The methodology used in the study for agro-drought monitoring was an approach to integrate remote sensing with GIS database on the basis of two indicators: SDI and MPDI. As described in above, SDI was computed from NDVI and LST, and MPDI from precipitations over previous eight periods. Combination of the two indicators could be done as follows (Qin et al. 2005) [17]:

(7) $$\text{SADI}=B_{1}\times \text{SDI}+B_{2}\times \text{MPDI}$$

where SADI was the comprehensive index for agro-drought monitoring in Guangdong, *B*_1_ and *B*_2_ were the weighs of SDI and MPDI, respectively. Since remote sensing provided more details of spatial information than the limited meteorological observation stations, we might give more weight to SDI than to MPDI. With this consideration, the values of *B*_1_ and *B*_2_ could be arbitrarily defined as *B*_1_= 0.6 and *B*_2_=0.4. After computation of SADI, classification of drought severity had to be done in order to make the results of drought monitoring more understandable for public and administrations. Five grades had been commonly used to separate various severities of drought in Guangdong and China: severe drought, moderate drought, slight drought, normal condition, and wetness. There was no drought for agriculture when the grade of normal condition was observed. And the fields had sufficient soil water supply for crop growing when the grade was under the wetness. According to this common classification of drought severity, the value of SADI for a pixel could be divided into the following five grades: 1 to 15 as severe drought, 15 to 30 as moderate drought, 30-50 as slight drought, 50-70 as normal condition, and 70-100 as wetness (Qin et al. 2005) [17].

### 2.9 Image processing procedures in drought monitoring

**Figure 3.**
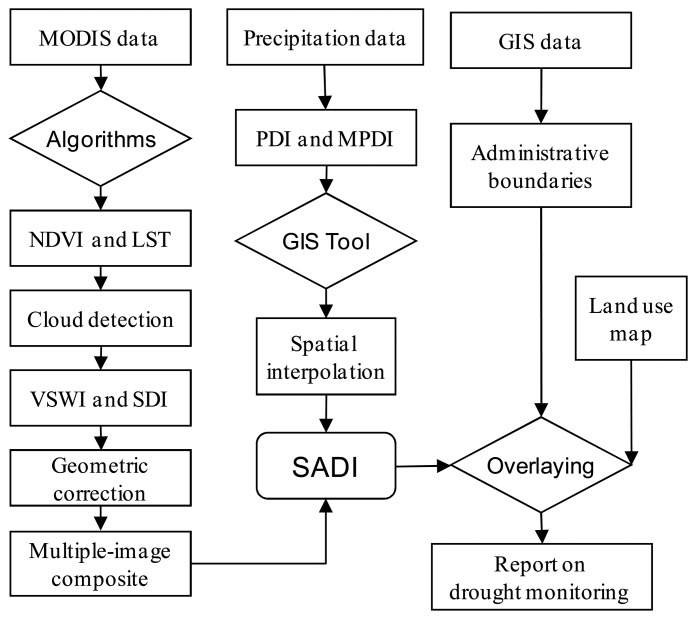
Procedures of the approach for agro-drought monitoring in Guangdong.

The approach developed in the study for drought monitoring mainly involved a procedure of 3 steps. The first step was to compute the drought indicator SDI from remote sensing data, which might include a series of computation processes including LST retrieval, NDVI and VSWI calculation, and geometric correction. The second step was to compute MPDI from available GIS database of meteorological data, which might include the processes of computing historical average rainfalls and carrying spatial interpolation of MPDI into the entire region of Guangdong province. Finally the third step was to integrate the above two indicators under GIS framework to classify grades of drought severity to produce the results of agro-drought monitoring for the current 10-days period, which might include the multiple-images composition and mutual correction to fit into a fixed mapping projection. As remote sensing data are usually affected by cloud or other factors, the computation of SDI indicator may have errors in some pixels. Consequently it was necessary to construct a GIS database to involve the data on terrain and land cover conditions for agro-drought monitoring and assessment. Operation of the procedure could then generate the maps of agro-drought monitoring for each 10-days period in the province. Figure 3 showed the flow chart for data processing procedure used in the study.

## 3. Results and Analysis

### 3.1 Spatial distribution of agro-drought in Guangdong

**Figure 4.**
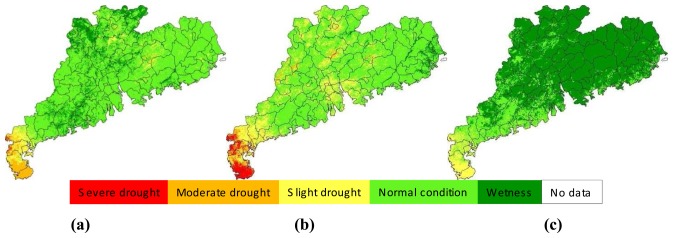
Drought monitoring results in early-May **(a)**, Mid-May **(b)** and late-May **(c)**, 2006 in Guangdong.

**Figure 5.**
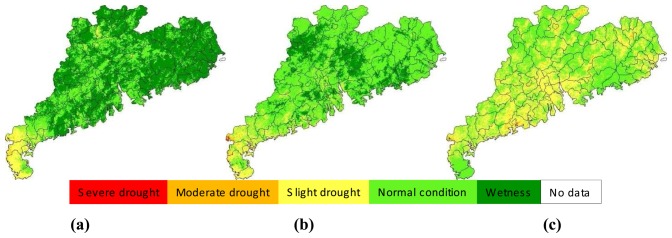
Drought monitoring results in early-June **(a)**, Mid-June **(b)** and late-June **(c)**, 2006 in Guangdong.

**Figure 6.**
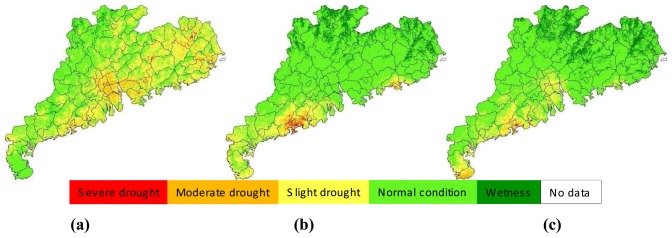
Drought monitoring results in early-July **(a)**, Mid-July **(b)** and late-July **(c)**, 2006 in Guangdong.

**Figure 7.**
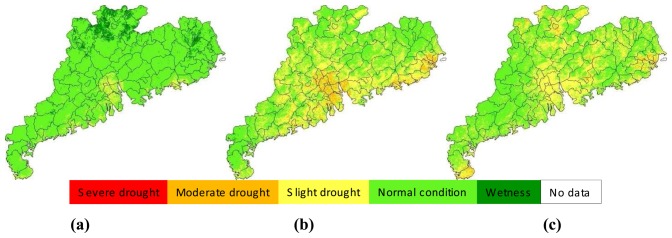
Drought monitoring results in early-August **(a)**, Mid- August **(b)** and late-August **(c)**, 2006 in Guangdong.

**Figure 8.**
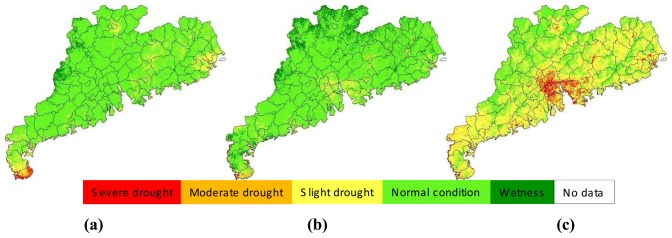
Drought monitoring results in early-September **(a)**, Mid-September **(b)** and late-September **(c)**, 2006 in Guangdong.

Agro-drought monitoring in Guangdong in 2006 was carried out using the proposed approach in the study. Figures 4, 5, 6, 7 and 8 showed the results of agro-drought monitoring during the main growing seasons between May and September 2006, which were a series of maps indicating drought severity variation in each 10-days period. The growing seasons of main crops such as rice and maize in Guangdong were usually from March to October. Thus the results shown in Figures 4, 5, 6, 7 and 8 represented the principle information of soil moisture conditions for agriculture during the growing seasons. As shown in Figure 4, drought in May 2006 was not severe in Guangdong. Moderate to slight drought was seen in Leizhou Peninsula in the west in early-May. The dryness condition in the peninsula became severe in the mid-May period due to little rainfall. The general soil moisture condition in the province was also shifting toward drying process in the mid-May period, as seen in Figure 4b. The soil moisture condition became normal in the late-May period as a result of several rains (Figure 4c).

The drought situation in June was more or less similar with that in May. In early-June, most parts of the province were under normal to wet condition for agricultural cropping (Figure 5a). This was because several rains during the period and the continuous impact of cropping condition from late-May, which was generally wet for the entire province. Due to decrease of rainfall, drought situation was moving into drying in the mid-June period (Figure 5b) and a general slight drought was observed in many places of the province (Figure 5c). This drying process was aggregated in the next period of early-July (Figure 6a). However, the drought was relieved in the mid-July (Figure 6b) and this relief into general wet condition was extended into late-July and early-August (Figures 6c and 7a). It could be said that the surface condition was under the best moisture for agriculture in early-August because almost the entire province was under normal-to-wet condition (Figure 7a). As rainfall decrease and continuous evapotranspiration, the ground in mid-August and late-August periods (Figures 7b and 7c) changed again into drying conditions until early-September (Figure 8a). The general wet condition in almost entire territory of Guangdong seen in early-September was consecutively extended into mid-September period (Figure 8b). Locating in subtropical region and bordering the west Pacific ocean, Guangdong had a climate of monsoon-dominated pattern, which was characterized with rainfall concentrating in the summer, leading to very few rains in autumn. As the autumn coming in late-September, the ground in the province was under a rapid drying process and the slight drought was observed in many places of the province (Figure 8c). This drying process usually lasted for several months until next growing season.

**Figure 9.**
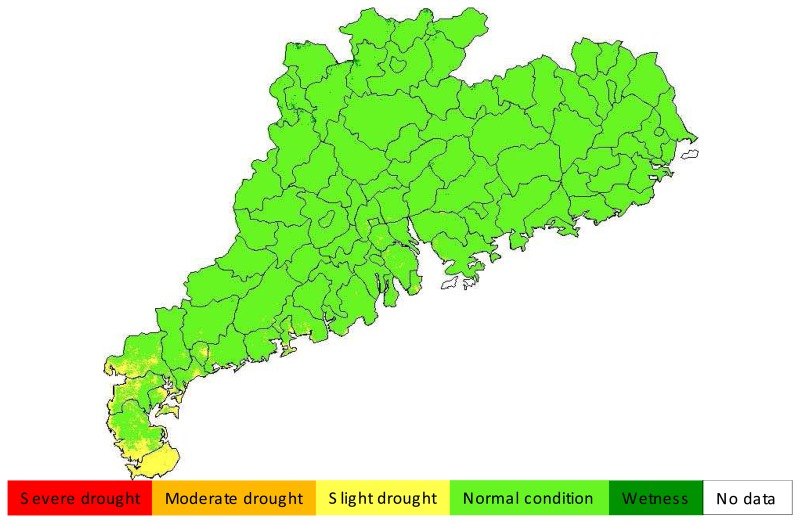
Average drought severity during May and September 2006 in Guangdong.

In order to have an overall assessment of drought situation in 2006, we carried on an average of the monitoring results for the periods from May to September, which gave the result shown in Figure 9. It could be said that the general situation of ground surface in 2006 was very suitable for agricultural cropping in Guangdong. The grade of drought severity was normal-to-wet condition for most parts of the province (Figure 9). Only in Leizhou Peninsula slight drought was observed. This probably could be attributed to the relatively small amount of rain in the peninsula in 2006.

### 3.2 Seasonal variation of agro-drought in 2006

**Figure 10.**
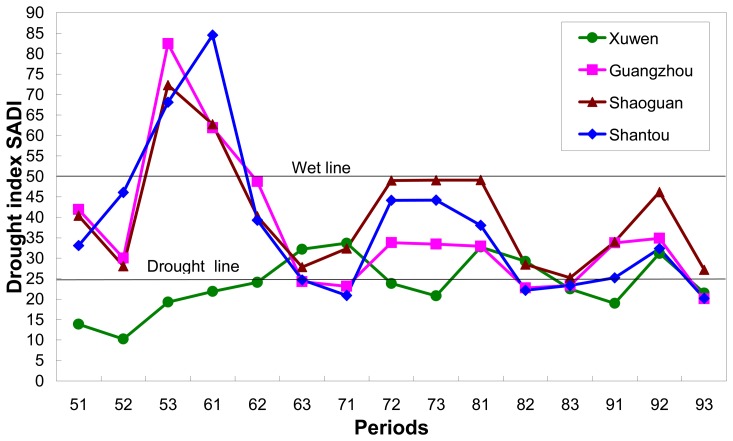
Seasonal variation of agro-drought in selected regions of the province in 2006.

Drought grade in Guangdong also displays a great difference among seasons. Four regions were selected to compare the seasonal change of drought severity in 2006: Xuwen in the west Leizhou Peninsula, Guangzhou in the middle Zhujiang Delta, Shaoguan in the north mountains and Shantou in the East coastal region (Figure 1). The drought index of the four typical regions was extracted from the results of every 10-days period shown in Figures 4, 5, 6, 7 and 8. Seasonal variation of the drought index of the four regions was shown in Figure 10, in which the drought line and wet line was also showed. The result in Figure 10 confirmed the generally favorable condition of ground surface for agricultural cropping in the province for growing seasons. However, seasonal changes were also obvious as to the degrees of favorable condition. The highest value of drought index was observed in the periods of late-May and early-June in Guangzhou, Shaoguan and Shantou. As a contrast, the lowest value of the index was also seen in this period in Xuwen. Since the higher the index, the wetter the ground surface is. Therefore, we could observe a sharp difference in drought severity in the province during this season. As shown in Figure 10, the ground was generally in normal condition for agriculture during the growing seasons ranging from May to September. The slightly worse conditions were observed in the periods between late-June and early-July, as well as between mid-August and early-September. As a contrast, the general better conditions appeared in the period between mid-July and early-August. Since the drought index of the four regions located above the drought line, it could be concluded that drought in 2006 was not severe in Guangdong even though great variation of the drought index was observed among the seasons (Figure 10).

### 3.3 Comparison of SDI and MPDI for drought monitoring

**Figure 11.**
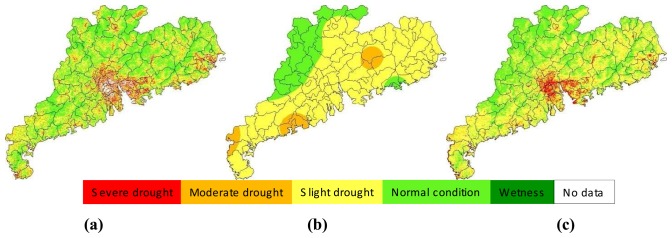
Drought monitoring results in late-September, 2006 in Guangdong, indicated by SDI **(a)**, MPDI **(b)** and SADI **(c)** respectively.

The approach developed in the study for drought monitoring was a combination of remote sensing and meteorological data. Thus it was necessary to compare the results from the two data. Figure 11 presented an example of illustrating the comparison for the late-September period, with Figure 11a showed the result of drought monitoring with remote sensing through SDI index and Figure 11b the result from meteorological data through MPDI index. Since SDI represents the drought severity indicated by the normalized vegetation supply water index calculated from remote sensing data, the result shown in Figure 11a represents the general condition of drought severity observed from space. As seen in Figure 11a, moderate-to-severe drought was seen to happen in the middle Guangdong region, especially the Zhujiang Delta, with Guangzhou having an average drought index of about 20% (Figure 10). MPDI represents the drought severity indicated by the normalized precipitation distance index calculated from precipitation data (Figure 11b). The results showed in Figure 11c was the combination of SDI and MPDI to form a comprehensive drought index SADI as the final drought monitoring result for the monitoring period, i.e. late-September in Figure 11.

As shown in Figure 11a, drought was also severe in eastern Guangdong, with Shantou having an average drought index of 18%. This was consistent with spatial distribution of precipitation shown in Figure 11b as MPDI index. The general drought observed in this period was attributed to the little rain. As indicated by MPDI, the most parts of Guangdong except the small region in northwest part were under slight-to-moderate drought as a result of little rain in the period. Leizhou Peninsula the most serious drought in other period was observed to have similar condition with other regions in this period. Therefore, combination of these two data gave a reasonable result of drought monitoring shown in Figure 11c. The severe drought observed in the middle Zhujiang Delta was mainly attributed to the severe drought index computed from remote sensing data.

### 3.4 Comparison with rainfall distribution

**Figure 12.**
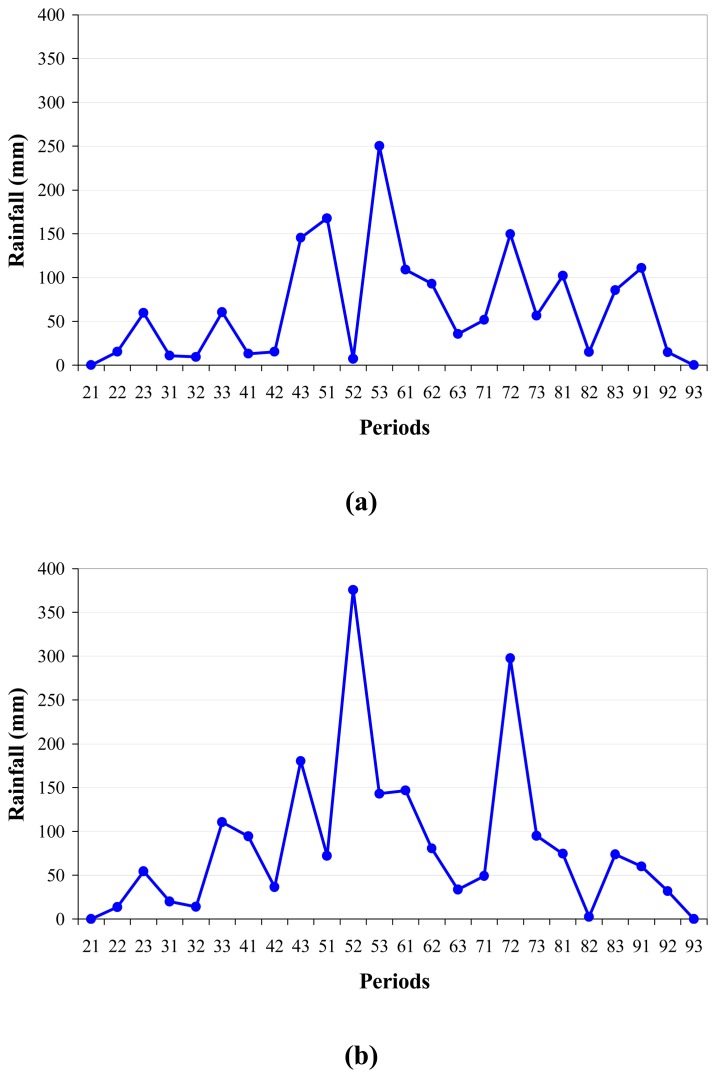

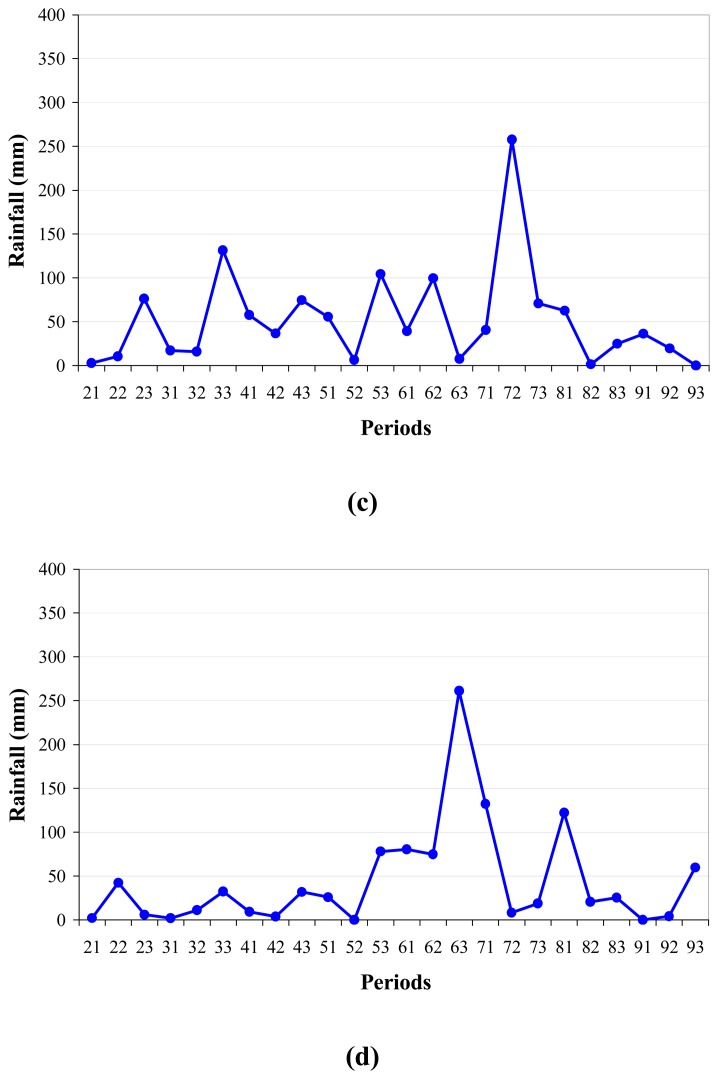
Comparison of precipitation in four typical sites, with **(a)** Guangzhou in the middle, **(b)** Shantou in the east, **(c)** Shaoguan in the north and **(d)** Xuwen in the west **(d)**. The label 51 in period axis denotes the period early-May, 62 the period mid-June, 73 the period late-July, and so on (the 1^st^ digit as month and the 2^nd^ as period).

Altitude of terrain in Guangdong is generally higher in the north than in the south. Most of the northern Guangdong is featured with mountainous landscape. In order to interpret the spatial distribution of drought severity in the province, four typical meteorological stations with rainfall data in each period of 2006 were selected to compare with the monitoring results. Figure 12 shows the seasonal variation of precipitation in the four stations during the growing period from May to September. The selected stations were Guangdong in the middle, Shantou in the east, Shaoguan in the north and Xuwen in the west. As seen in Figure 12, precipitation was generally higher in Guangzhou and Shantou than in Shaoguan and Xuwen. Numerically the annual precipitation of the four stations was 1578.1mm in Guangzhou, 2060.0mm in Shantou, 1248.6mm in Shaoguan and 1051.7mm in Xuwen. These differences in precipitation explained the spatial variation of drought in Guangdong. Since the east had the highest precipitation, it had the lowest drought severity in the growing period in 2006, as shown in Figure 9. The general wet condition seen in the middle and the north also was with accordance with the relatively sufficient rainfall in these two regions. The relatively severe drought found in Leizhou Peninsula of western Guangdong was mainly due to its small amount of rainfall.

Specifically, in Guangzhou, the high peak of rainfall appeared in late-May while the lowest amounts of rainfall were seen in mid-May, mid-August and late-September (Figure 12a). Consequently these periods were seen to have relatively higher severity of drought as shown in Figures 4b, 7b and 8c. Though we did not carry on monitoring campaign for the period from February to April, the low precipitation in these months (Figure 12a) should produce a severe drought condition in the middle region centering at Guangzhou. The amount of precipitation was relatively high in the east represented by Shantou station (Figure 12b), where only in mid-August was seen to have little rainfall, leading to the severe drought observed in the period (Figure 7c). As a contrast, small amount rainfall shown in Figures 12c and 12d corresponded to the relatively more serious drought condition in the west and the north of the province. In Xuwen little rain was seen in the period before late-May, leading to the severe drought in early-May and mid-May observed in Figures 4a and 4b. The sufficient rain did not appear in Xuwen until late-June. In the periods after mid-August, the rain in Xuwen was also not enough to provide a normal condition for agriculture, leading to a general drought phenomenon in the region. Another important feature shown in Figure 12 was the similar pattern of precipitation in Guangdong even though the exact time of the peak might be difference in different places, leading to spatial and seasonal heterogeneity of soil moisture conditions for agricultural cropping in the province.

### 3.4 Comparison with other sources

**Figure 13.**
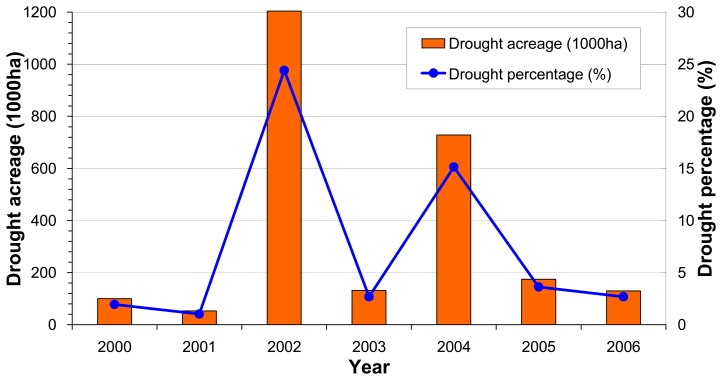
Drought acreage and its percentage to total cropping acreage in Guangdong in recent years.

In order to validate our monitoring results, we checked the reported drought acreage in statistics. Unfortunately we only found the total drought acreages of the province in each year and had no data on the spatial distribution of the drought events. However, these data were still able to provide us a rough comparison with our monitoring results from the methodology developed in the study. As shown in Figure 13, agricultural drought disaster in 2006 was not serious in Guangdong due to sufficient rainfall in the growing seasons. Total drought acreage was only statistically 130 thousand ha in 2006, accounting for only 2.68% of the province's total cropping acreage (MOA, 2007) [18]. As a contrast, agricultural drought was very severe in 2002 when there was 1204 thousand ha of cropping area suffering from drought attacks, with a drought percentage of 24.41% (Figure 13). The small drought acreage and its percentage to total cropping acreage in 2006 explained why we did not obtain a severe drought scenario (Figure 9) from our monitoring campaign in this study. Therefore, it could be concluded that the low drought obtained in our study was exactly coincident with the low statistics of drought disaster in the province in 2006.

**Figure 14.**
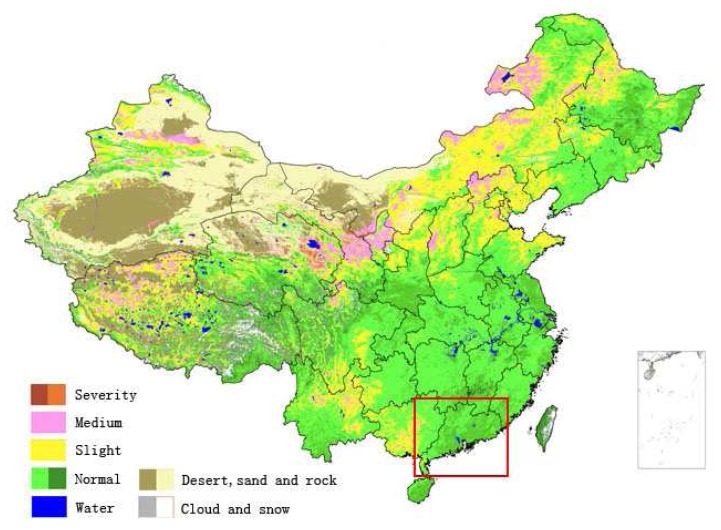
CNSMC's result of drought monitoring for entire China in mid-May 2006, with red rectangle showing geographical location of Guangdong and its adjacent regions.

Another comparison was with the reported drought monitoring result from Chinese National Satellite Meteorological Center (CNSMC) [19], which used another independent method to compute drought severity from remote sensing data (mainly NOAA-16 and Chinese meteorological satellite FY). The CNSMC issued their monitoring results of the entire China according to their method. The monitoring results of CNSMC were also presented in a format of 10-days period. For simplicity, only one scene of the drought monitoring results from CNSMC was used to compare with our result. Figure 14 showed the result of CNSMC for mid-May period of 2006 for entire China. Red rectangle in Figure 14 illustrates the region of Guangdong and its adjacent areas. Comparison of Figure14 with our corresponding result for the same period of mid-May (Figure 4b) indicated that the spatial distribution of drought severity had a very high similarity between the two. Both results highlighted that drought severity was very low in the province. Most places of Guangdong were in the grades of normal-to-wet condition for agriculture. Only Leizhou Peninsula in the west Guangdong was found to have the sign of drought. But the severity of drought in the peninsula was given in slight difference between the two results, with higher severity in ours than in CNSMC's. The peninsula was monitored as severe drought in our result (Figure 4b) while it was given as slight drought in the results of CNSMC (Figure 14). Considering the little rains for a long time before mid-May period in the peninsula as shown Figure 12d, we believed that our monitoring result of classifying the peninsula as the grade of severe drought instead of slight drought was a correct decision. This slight difference in presenting drought severity validated that our study provided a better approach to monitor agro-drought from both remote sensing and precipitation data with help of GIS spatial analysis than only from one source of remote sensing in Guangdong.

## 4. Conclusions

A practical approach was developed in the study for agro-drought monitoring in Guangdong. The approach was based on vegetation supply water index (VSWI) computed from MODIS remote sensing data and precipitation distance index (PDI) computed from ground-observed precipitation data. A comprehensive index for assessment of agro-drought severity (SADI) was then established from the normalized VSWI and PDI under the assistance of GIS spatial analysis. The value of SADI was within the range of 0 to 100, with 0 representing the most serious drought and 100 the wettest condition for agriculture. Five grades of drought severity were then identified to indicate various degrees of drought severity: severe drought, moderate drought, slight drought, normal condition and wetness.

The approach was applied to Guangdong for drought severity monitoring in the main growing seasons from May to September, which generated a series maps showing spatial distribution of drought severity for every 10-days period in the province. The monitoring results showed that the drought severity on average was very low in 2006 in Guangdong. However, seasonal variation of the severity was also obvious in difference places of the province. Higher severity of drought could be seen in the periods of late-June, early-July, mid-August and late-September. Regionally, Leizhou Peninsula in the west had the most serious drought in the periods before mid-May. Zhujiang delta in the middle suffered from moderate drought in the periods of mid-August and late-September. Shaoguan in the north had slight drought in the periods of late-June, mid-August and late-September. Both spatial and seasonal patterns of drought severity were well coincident with the precipitation in the province.

In order to validate our monitoring results, comparisons had been done with the drought statistics in recent years and the drought monitoring results from CNSMC, which used only remote sensing data for their monitoring. The general consistence of our results from both drought statistics data and CNSMC's results confirmed the applicability of our approach for drought monitoring. Our relatively better identification of drought severity in Leizhou Peninsula of western Guangdong than that of CNSMC may suggest that the approach developed in the study is able to provide a better alternative to increase the accuracy of drought monitoring for agricultural administration and farming. The approach may also be applicable to other regions for drought monitoring in the case of available data required.

## Figures and Tables

**Table 1.** Determination of *VSWI_d_* and *VSWI_W_* for drought monitoring in Guangdong.

NDVI	VSWI_d_	VSWI_W_	NDVI	VSWI_d_	VSWI_w_
<0.05	0.11	0.25	0.35-0.4	0.89	2.0
0.05-0.1	0.22	0.5	0.4-0.45	1.0	2.25
0.1-0.15	0.33	0.75	0.45-0.5	1.11	2.5
0.15-0.2	0.44	1.0	0.5-0.55	1.22	2.75
0.2-0.25	0.56	1.25	0.55-0.6	1.33	3.0
0.25-0.3	0.67	1.5	0.6-0.65	1.44	3.25
0.3-0.35	0.78	1.75	0.65-0.7	1.56	3.5
